# Egg donors’ motivations, experiences, and opinions: A survey of egg donors in South Africa

**DOI:** 10.1371/journal.pone.0226603

**Published:** 2020-01-15

**Authors:** Donrich Thaldar

**Affiliations:** School of Law, University of KwaZulu-Natal, Durban, South Africa; University of Toronto, Rotman School, CANADA

## Abstract

The objective of this study was to gain information from egg donors in South Africa (SA) which could be pertinent to policy development on egg donation. The study was conducted on egg donors in the database of a Cape Town-based egg donation agency who donated within a year preceding the study. 150 egg donors from the population of 226 participated in an online survey. The main results are: 95% of respondents experienced egg donation as being positive. However, 7% of respondents report not giving proper informed consent, and a similar percentage of respondents also report not knowing whether any medical risks actually materialised as sequelae to their donations. This is a cause for concern and should be investigated further. Regarding donor anonymity, which is currently the legal position in SA, 79% of respondents indicated that they would still have donated had they been legally required to release their identities. Accordingly, possible legal reform away from the current system of donor anonymity seems unlikely to significantly impact the supply of donated eggs. Regarding motivation, respondents report being primarily motivated by wanting to help infertile women. However, respondents believe that a fair and realistic amount of compensation would be about 60% higher than what is currently paid as the national standard fixed amount. This fixed-amount compensation system should be further investigated in terms of its legality, impact on donor profile, and its current amount.

## Introduction

While there has been much research on egg donors’ motivations, experiences, and opinions in other areas of the world–especially in the United States and the United Kingdom [1,2]–no such research has thus far been conducted in South Africa (SA). The present study aims to fill this lacuna by presenting the results of a quantitative survey of SA egg donors (the ‘SA survey’).

Assisted reproduction–and egg donation in particular–are highly regulated in SA. The central regulatory instruments are the National Health Act [3], and the Regulations Relating to Artificial Fertilisation of Persons [4] made by the Minister of Health in terms of the Act. Read together, the Act and the Regulations outlaw trade in human gametes, and provide that gamete donors can only be reimbursed for their reasonable expenses. What exactly would qualify as ‘reasonable expenses’ is however unclear, and has not as yet been tested in the courts. In any event, this legal provision is not applied in practice. A private association of healthcare professionals in the fertility industry, the Southern African Society of Reproductive Medicine and Gynaecological Endoscopy (SASREG), determined R7,000 as the upper limit for payment to egg donors (S2 Document). Their stated rationale for this amount is that it compensates egg donors not only for expenses (as allowed by law), but also for time, inconvenience, physical and emotional demands, and risks associated with egg donation (S1 Document). This SASREG-determined upper limit amount of R7,000 per egg retrieval has, in practice, become the standard *fixed-amount payment* to egg donors.

Although the Regulations Relating to Artificial Fertilisation of Persons limit the number of children that may be conceived from a single donor to six, there is no limit on the number of times that a donor can donate, or on the number of eggs that she can donate. Accordingly, because not all fertilised eggs will be viable or will lead to a successful pregnancy, it is conceivable that egg donors may be donating multiple times.

There are no statutory provisions regarding advertising to recruit egg donors. However, SASREG’s guidelines (S1 Document) provide that advertising and publicity materials should avoid making statements about ‘earning money’ or ‘financial gain’, but may refer to ‘reimbursements’ or ‘compensation’.

Regarding age restrictions on egg donation, the National Health Act provides a minimum age– 18 years–but no maximum age. The SASREG’s guidelines (S1 Document) stipulate 18 years as a minimum age, and 36 as a maximum age, but allow for exceptions. These guidelines further state that egg donors should ‘preferably’ be between 20 and 35 years of age.

A further salient aspect of SA’s gamete donation regulatory regime is donor anonymity. The SA’s Children’s Act [5] provides that while donor-conceived children have the right to access all medical information about their genetic parents–the gamete donors–the identity of the donors may not be released. However, this does not exclude the use of known donors–women known to the intended parents prior to egg donation. Both the Regulations Relating to Artificial Fertilisation of Persons [4] and SASREG’s guidelines (S1 Document) make reference to known donors. The SA Law Reform Commission [6] is currently investigating whether donor-conceived children should have a right to know the identity of their donors, as has been recognised in other jurisdictions like the United Kingdom.

The present study is the first to give a voice to SA egg donors on these regulatory issues, and other issues pertinent to public policy development relevant to egg donation. I investigated a broad range of questions relating to egg donation in SA: (1) What is the demographic profile of egg donors? (2) How many times do egg donors donate? (3) How do egg donors perceive the quality of their genetic contribution to posterity? (4) What motivates egg donors to donate? (5) Do egg donors generally engage in the act of giving in the healthcare context? (6) Are the medical risks adequately explained to egg donors? (7) Are egg donors satisfied with having donated eggs? (8) How frequently do any of the medical risks actually materialise? (9) What are egg donors’ opinions about donor anonymity versus donor identity release? (10) What are egg donors’ opinions about financial compensation for egg donation?

## Materials and methods

Ethical approval was obtained from the Humanities and Social Sciences Research Ethics Committee of the University of KwaZulu-Natal. (Protocol reference number HSS/1089/017, as amended.)

### Participants

Permission was obtained from Nurture, a Cape Town-based egg donor agency, to access its database of 226 egg donors who donated from September 2017 until August 2018. First, Nurture emailed these donors to inform them of the survey, and to give those who did not wish to participate, the option to opt out. Two donors opted out. Subsequently, during the last week of September 2018, the remaining 224 egg donors were emailed by the author and invited to participate in the study by completing an online survey. The email message contained a ‘Begin survey’ link that, if clicked, would open the online survey in the respondent’s internet browser. An unsubscribe link was also provided. By clicking the ‘Begin survey’ link, the respondent indicated and electronically recorded her consent to participate in the study. A reminder email was sent during the first week of October, and again during the second week of October–after which the survey was closed during the third week of October. All emails to the donors stated that their identities would be kept confidential. Of the population of 224 egg donors who were contacted, 150 took the survey, and 7 unsubscribed. This exceeded the minimum sample of 143 required to represent the population of 226, using an alpha level of .05 and a margin of error of .05. Answering all the questions in the survey, however, was not compulsory–a respondent could proceed to the next question without having to answer a question. As such, some questions were answered by fewer than 150 respondents. Most of the questions received 149 or 150 responses. Only in the question on donor motivation, the number of responses drop below the minimum representative sample size of 143. The number of respondents who answered each question is indicated in the Results section below.

### Measures

#### Demographic profile

A basic demographic profile of the respondents with reference to educational level, race, employment status, and income was established. With educational level, the respondents were asked to indicate their highest educational qualification from four options: ‘Grade 10’, ‘Grade 12’ (secondary school completion), ‘College diploma’ (which is at Grade 10–12 level, but would typically be industry-specific or vocational skills training), and ‘University degree’. With race, the SA standard classifications of ‘Black African’, ‘White’, ‘Coloured’ (a multiracial ethnic group in SA), ‘Indian’ and ‘Other’ were used. Employment status included ‘Employed full-time’, ‘Employed part-time’, or ‘Unemployed/full-time student’. Regarding income, respondents were requested to enter their monthly income.

#### Genetic self-perception

This question aimed to ascertain how egg donors perceived the quality of their own genetic contribution to posterity–essentially to develop a ‘genetic self-perception profile’ of the respondents. As such, donors were asked their opinion on the statement: ‘I have good genes’. Response options were: ‘Strongly agree’, ‘Moderately agree’, ‘Slightly agree’, and ‘Disagree’. The statement ‘I have good genes’ is open for subjective interpretation. However, this is in line with the purpose of this question, namely to determine the donors’ subjective genetic self-perceptions.

#### Number of donations

Do donors typically donate only once, or do they donate repeatedly? Respondents were asked to enter the number of times that they had donated eggs.

#### Motivation for donation

Studies of donor motivation often use the terminology ‘altruism’ in juxtaposition with financial motivation. On the other hand, some studies recognise that there is not necessarily a binary choice, and have attempted to allow for a mixture between altruism and financial motivation. However, this leads to the illogical result that a donor can be selfless (altruistic) and selfish (financially motivated) at the same time. This paradox is solved if the concept ‘altruism’ is replaced with ‘wanting to help’, given that ‘wanting to help’ contains the same element of concern for the wellbeing of others as altruism, but without the necessary element of selflessness.

Donor motivation was investigated using three metrics: wanting to help, financial gain, and genetic legacy. Respondents were requested to rate the importance of each one of these three metrics. The question was posed: ‘Why did you decide to become an egg donor?’ The three possible answers were: ‘I wanted to help infertile women’, ‘I wanted to earn money’, and ‘I wanted to pass on my genes to a child’. The rating scale for each one of these answers included the following options: ‘Very important’, ‘Important’, ‘Slightly important’, and ‘Not important’.

Although ‘altruism’ and financial gain are the two most frequently cited motivators for donation [7], a less frequently cited motivation, namely genetic legacy (‘I wanted to pass on my genes to a child’) was also included. Although Kalfoglou and Gittelsohn [8] refer to wanting to pass on one’s genes as an ‘unconventional’ motivation, a study of online sperm donors in the United Kingdom by Freeman et al. [9] shows that this is, in fact, an important motivation.

#### Other instances of giving

This question investigated whether the respondents are involved in other acts in the healthcare sphere that also entail giving something from one’s own body to be used by somebody else–namely blood donation and being a registered organ donor. In SA there may be no compensation at all for these acts.

#### Medical risk: Informed consent and incidence of complications

Next, informed consent was investigated by asking the question: ‘Prior to donating your eggs, did the medical practitioner who performed the egg donation procedure inform you that there is a small chance of medical complications?’ Response options were ‘Yes’, ‘No’, and ‘Unsure’. Related to informed consent, is the question whether any medical complications actually occurred. The question was asked: ‘Were there any medical complications due to your egg donation(s)?’ Again, response options were ‘Yes’, ‘No’, and ‘Unsure’.

#### Satisfaction with egg donation

The question asked was: ‘Are you glad that you became an egg donor?’ The response options were ‘Yes’, ‘No’, and ‘Unsure’.

#### Donor identity release

Two questions were posed to probe respondents’ opinions on the possibility of donor identity release. The first question envisaged a new legal regime in which a donor has a choice as to whether she wants to be anonymous or to release her identity. The question read: ‘Currently, the law provides that egg donation must be anonymous. If the law was different, namely to make it the egg donor’s choice whether to release her identity to the child who was conceived with the donated eggs, what would you have decided?’ Three response options were provided: ‘I would have chosen to remain anonymous’, ‘I would have chosen to release my identity to the child who was conceived with my donated eggs, if the child wanted to know’, and ‘I don’t know’.

The second question envisaged an alternative, new legal regime in which a donor does not have a choice, but in which identity release is compulsory. This question was formulated as follows: ‘Imagine a different scenario: If the law was completely different, namely to require an egg donor’s identity to be released to a child who was conceived with the donated eggs, if the child wanted to know, would you still have become an egg donor?’ The response options were ‘Yes’, ‘No’, and ‘Unsure’.

#### Donor remuneration

Lastly, respondents’ opinions regarding fairness in the context of compensation for egg donation were investigated. First, respondents’ views of the current legal regime of cost-recovery were probed with the question: ‘Currently, the law provides that egg donors may only be compensated for reasonable costs incurred, and they do not receive any other or further payment. What is your opinion on this legal provision?’ Response options were: ‘I think it is fair’, ‘I think it is unfair’, and ‘Unsure’. Secondly, respondents were requested to enter an amount of compensation for their egg donation that, in their opinion, would have been fair and realistic.

## Results and statistical analysis

### Notes on statistical analysis

Responses were downloaded from the online survey platform as a spreadsheet and anonymised. Given the exploratory nature of the paper, the statistical analysis is largely descriptive, and is complemented with inferential analysis where relevant. To test if one response option was selected significantly more often than the others, a binomial test was applied to questions with two possible responses; while a chi-square goodness-of-fit test was used when a question had more than two response options. The chi-square test of independence was used to test for significant relationships between two categorical variables. ANOVA was used to test for differences in an interval variable (e.g. average income) across two or more independent groups. Pearson’s correlation was used to explore linear relationships between two interval measures. A paired t-test was applied to test for significant differences in two related interval measures, e.g. payment that is deemed fair and income.

### Demographic profile

The respondents’ demographic profile is presented in Table 1. The modal respondent was white, her highest qualification was Grade 12, and she was employed full-time. There was no significant difference in the educational profiles for full-time and part-time employed respondents. The average monthly income of all respondents was R12,054. Table 2 compares the income profile of full-time employed respondents with part-time employed respondents. Six respondents did not provide information on their income (two who were employed full-time; three who were unemployed or studying full-time; one who did not specify her employment status). The twenty respondents who indicated their income as R0 were all unemployed or studying full-time. Feedback on the income question that approximates to R0, like ‘none’ or ‘N/A’ (not applicable), was taken as R0.

**Table 1:** Demographic profile of respondents.

Variable	n	%
***Race***		
Black African	49	32.7
White	79	52.7
Coloured	13	8.7
Indian	3	2
Other	5	3.3
Unspecified	1	0.7
***Highest qualification***		
Grade 10	1	0.7
Grade 12	63	42
College diploma	38	25.3
University degree	47	31.3
Unspecified	1	0.7
***Employment***		
Employed full-time	86	57.3
Employed part-time	21	14
Unemployed/studying full-time	42	28
Unspecified	1	0.7

**Table 2:** Monthly income of respondents.

	All respondents	Employed full-time	Employed part-time
**n**	144	84	21
**Mean**	R12,054	R16,455	R13,143
**Median**	R10,000	R15,000	R8,000
**Standard deviation**	R10,881	R9,124	R14,360
**Range**	R65,000	R53,000	R64,000

### Genetic self-perception

Regarding the statement ‘I have good genes’, 55% (n = 83) of respondents strongly agreed, 37% (n = 55) moderately agreed, 5% (n = 8) slightly agreed, and 2% (n = 3) disagreed; 149 respondents answered this question. Agreement to this statement was significantly moderate or strong, χ^2^ (3) = 119.107, p < .0005.

### Number of donations

Although all 150 respondents answered this question, 2 responses were clearly unrealistic (donated 18 and 60 times respectively) and were therefore excluded. One possible explanation is that these 2 respondents could have confused the number of *times* that they have donated with the number of *eggs* that have been retrieved from them during all their donations. Working with the remaining 148 responses, the results are as follows: The modal respondent had donated once, and the average number of times that a respondent had donated was 2.48. The highest number of times that a respondent donated was 7. The number of times that respondents had donated eggs is presented in Table 3.

**Table 3:** The number of times that respondents had donated eggs.

Variable	n	%
1	51	34,5
2	39	26,4
3	23	15,5
4	17	11,5
5	8	5,4
6	9	6,1
7	1	0,7

It was investigated whether there was an association between the number of donations and educational level, and between the number of donations and positive genetic self-perception–however, no significant association exists.

### Motivation for donation

**Fig 1:**
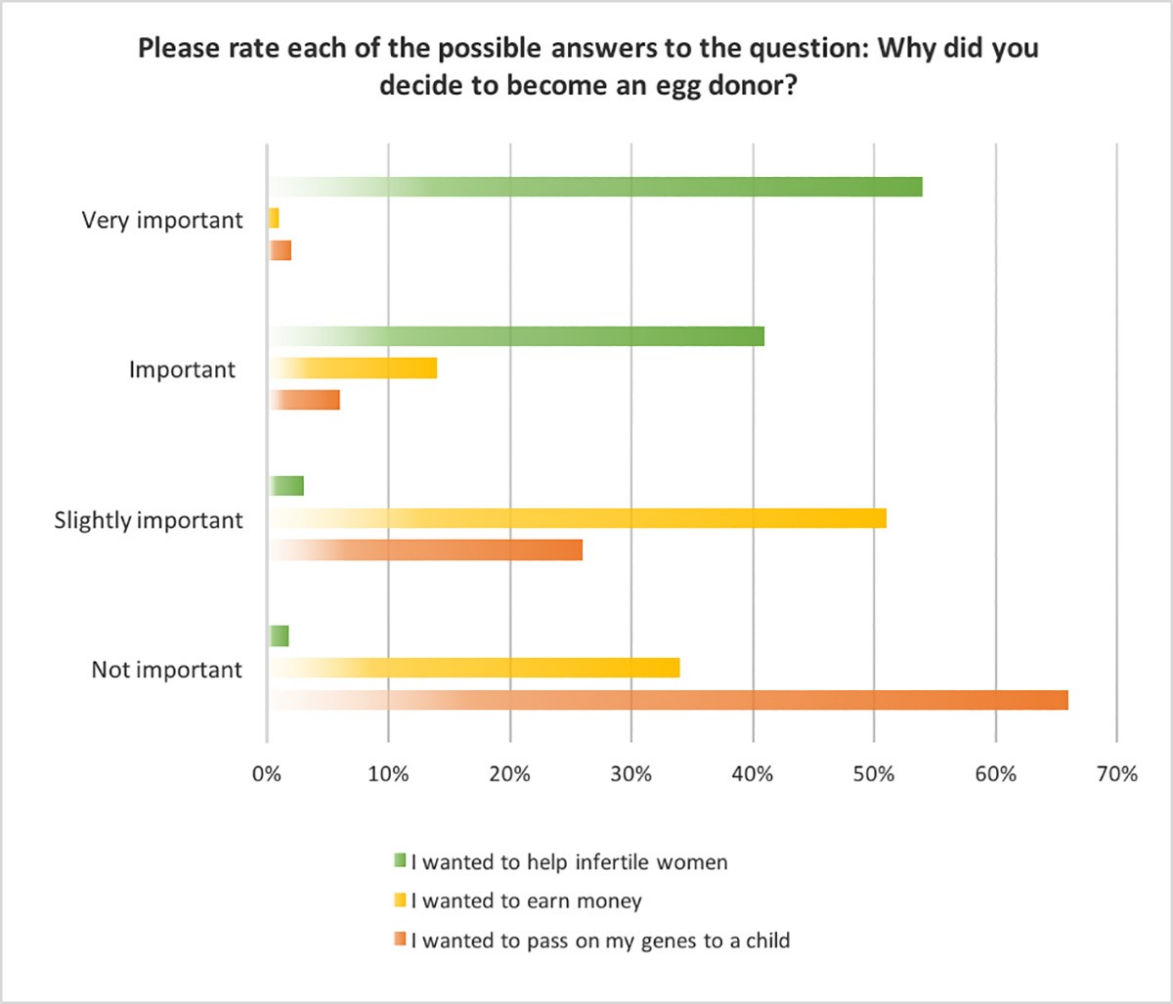
Rating of the three metrics for motivation for donation.

Wanting to help infertile women was clearly the strongest motivation for egg donation among the respondents, with a significant 95% (n = 137) of respondents who answered this question, ranking it as very important or important, χ^2^ (3) = 122.389, p < .0005. In contrast, financial gain and genetic legacy as motivations were significantly less important: of those who answered the questions, only 15% (n = 17) of respondents ranked earning money as either very important or important, χ^2^ (3) = 64.786, p < .0005; and a mere 8% (n = 10) responded that donating in order to pass their genes on was very important or important, χ^2^ (3) = 130.392, p < .0005. It should be observed, however, that two-thirds (66%, n = 74) of respondents did rank earning money as being at least slightly important. The importance allocated to each of the three metrics for motivation for donation that were provided as options, is illustrated in Fig 1. 144 respondents rated the wanting to help metric, 112 rated financial gain, and 125 rated genetic legacy. The percentage results are calculated as a proportion of the number of respondents who rated a particular metric.

### Other instances of giving

Just under half (47%, n = 70) of respondents were registered organ donors, and a significant number (70%, n = 105) have donated blood in the past, p < .0005. 149 respondents answered these questions.

### Medical risk: Informed consent and incidence of complications

On the question of informed consent, a significant 91% (n = 137) answered ‘yes’, while 7% (n = 11) answered ‘no’, and 1% (n = 2) were ‘unsure’, χ^2^ (2) = 227.880, p < .0005. On the question of whether there were any medical complications, a significant 88% (n = 131) answered ‘no’, 5% (n = 7) answered ‘yes’, and 7% (n = 11) were ‘unsure’, χ^2^ (2) = 199.946, p < .0005. 150 respondents answered the informed consent question, and 149 answered the medical complications question.

### Satisfaction with egg donation

A significant and overwhelming majority, 95% (n = 142), of respondents were glad they became egg donors, with 4% (n = 6) being unsure and only one respondent indicating that she was not glad, χ^2^ (2) = 257.732, p < .0005. 149 respondents answered this question. The respondent who indicated that she was not glad, experienced medical complications as a result of her donation, and is a one-time donor. Results from a chi-square test revealed that, compared to the donors who donated multiple times, a significant number of the one-time donors were either not glad or unsure about becoming an egg donor, χ^2^ (2) = 8.549, p = .007. Of the 6 respondents who indicated that they are unsure, all but 1 are one-time donors.

### Donor identity release

If SA law allowed egg donors to *choose* whether to be anonymous or to release their identities, a significant 54% (n = 81) would have chosen to release their identities, while 34% (n = 51) would have chosen to be anonymous, and the remaining 12% (n = 18) did not know, χ^2^ (2) = 39.720, p < .0005. In a scenario where the law *required* identity release, a significant 79% (n = 118) indicated they would still have become egg donors, 14% (n = 21) were unsure, and 7% (n = 11) would not have become egg donors, χ^2^ (2) = 139.720, p < .0005. Both of these questions relating to donor identity release were answered by 150 respondents.

### Donor remuneration

The current legal regime of limiting donor compensation to cost-recovery was perceived by a significant 69% (n = 103) of respondents as being fair, by 16% (n = 24) as being unfair, and 15% (n = 23) of respondents were unsure, χ^2^ (2) = 84.280, p < .0005. 150 respondents answered this question.

The amount of compensation for egg donation that the respondents deemed to be fair and realistic was on average R11,259, with a standard deviation of R10,004 and a mode of R10,000. 140 respondents answered this question. Although most respondents viewed the current regime of egg donor compensation as being fair in principle, the guideline by SASREG that effectively pegs egg donor compensation at R7,000 appears to be out of sync with egg donors’ perceptions of an amount that would constitute fair and realistic compensation. It was analysed if, on average, the R7,000 received per donation is significantly different from what the respondents think is a fair and realistic compensation, using a paired samples t-test for this. On average, the amount of R7,000 that donors received for a donation is significantly less than what they consider to be a fair and realistic compensation, t (139) = 5.037, p < .0005.

A paired samples t-test was used to investigate whether there is a significant difference between income and the amount of compensation deemed to be fair and realistic, but no significant difference exists. However, as can be expected, a respondent’s opinion on whether the current legal regime regarding donor compensation is fair or unfair, does impact on the amount of compensation deemed to be fair and realistic: Those respondents who believed the current legal regime regarding donor compensation was unfair, suggested a significantly higher amount for compensation, on average (R14,909), than those who believed the compensation to be fair (R9,156), p = .010.

Analysis using Spearman’s correlation showed that the amount that donors regarded as being fair and realistic compensation was positively correlated with their motivation to donate for financial gain. Higher financial motivation was associated with a larger amount considered as reasonable compensation, rho = .204, p = .035. A similar analysis on the relationship between a motivation of wanting to help and fair and realistic compensation amount showed no significant correlation.

## Discussion

### South African egg donors are positive about their experience

The finding of the SA survey that the overwhelming majority of respondents experienced egg donation in a positive way, is aligned with findings of studies elsewhere in the world. A meta-analysis of English-language research on egg donors [10] found that most donors have reported positive experiences of egg donation. In fact, a higher percentage of SA egg donors (95%) report satisfaction compared to their American counterparts (83%, as reported by Klock et al. [7], or 79%, as reported by Jordan et al. [11]).

As stated in the introduction, there are legal and SASREG-imposed age restrictions on egg donation in SA. This study did not gather data on its respondents’ ages. In a study of egg donors in Europe, Pennings et al. [12] found that there is a significant relationship between donor age and motives for donating. Accordingly, future research on egg donors’ motivations should include data on its respondents’ ages.

### Wanting to help stands out, but compensation cannot be ignored

Studies in other countries have shown that donor motivations correlate strongly with the legislative regime that governs egg donation in the specific country [13]. For instance, in the US where compensation for egg donation is market-driven, financial gain typically outranks ‘altruism’ as a motivation [14]. However, in European countries where compensation is limited or completely outlawed, ‘altruism’ dominates as a motivation [12]. SA’s de facto regime of a fixed amount of compensation is somewhere between the extremes of a market-driven system and a system that restricts compensation to expenses only. That said, the SA survey shows that wanting to help infertile women is by far the strongest motivation among SA donors. SA is not unique in yielding this result: A recent study in the UK, which also has a fixed-amount compensatory system, found that donors are primarily motivated by wanting to help infertile women [15].

It should however be noted that while wanting to help infertile women is the predominant motivation, financial compensation cannot be ignored. Earning money was at least of slight importance to almost two-thirds of respondents. Kenney and McGowan [14] observed that ‘altruism’ alone is insufficient to attract most donors. They based this observation on their own survey of US donors, and on the limited number of women who volunteer to donate in countries where compensation is severely limited or forbidden. It can therefore be argued that the fixed-amount system is a workable compromise between the public policy objectives of avoiding market-style commercialisation of egg donation, and still attracting donors. The fixed amount, which is justified as, among others, *compensation* for such unquantifiable concepts such as risk and discomfort, arguably acts as a financial *incentive*. This must be seen in the context that the average monthly income of the respondents (R12,054 for all; R16,455 for those who are full-time employed) is notably less than the average monthly income of people working in SA’s formal non-agricultural sector (R19,858, in February 2018) [16]. It would therefore appear that despite the fact that 61.6% of full-time employed respondents have a tertiary qualification, they are generally drawn from a population that earns about 17% below the country’s average income. As a consequence, even relatively low fixed amounts can serve as a sufficient financial incentive.

However, there is the group of respondents–just over one third–who indicated that receiving money is of no importance to them. Can these egg donors be described as altruistic in the true meaning of the word? Despite these donors’ low ranking of financial compensation, they still get paid the fixed amount. Should this cause one to be sceptical about the respondents’ response that receiving money is of no importance to them? Any such scepticism should be tempered by the fact that almost half of respondents were registered organ donors, and more than two-thirds had donated blood in the past. Given that less than 0.2% of the general population in SA are registered as organ donors [17], and less than 1% are blood donors [18], the high numbers of respondents who engage in these acts of giving something of their own bodies for the use of others in the healthcare context–without any financial reward–strongly support a conception of the typical egg donor as a person who has a general helpful disposition and whose helpfulness is not motivated by financial gain.

### Communication by medical practitioners: A red flag

The fact that 7% of respondents deny having given informed consent is a cause for concern. Similarly, the fact that a similar percentage of respondents were unsure whether there were medical complications, also raises concern. SA is not alone in this predicament: Kenney and McGowan [14], reporting on a study of 80 US egg donors, found that their respondents had a relatively poor understanding of the risks of egg donation. In fact, they observed that ‘a rather large and troubling minority (20%) of the respondents reported that they were unaware of any possible physical risks before initiating their first donation cycle’. In a study involving 25 egg donors in India, Jadva et al. [19] report that three respondents (12%) were not able to report what their eggs were used for.

The caveat with interpreting the responses to the questions on informed consent and the incidence of complications, is that the data were collected 1 to 12 months after donation, and that the study is therefore subject to recall bias. Note, however, that the studies by Kenney and McGowan [14] and Jadva et al. [19] were also retrospective. It is suggested that although recall bias may take the edge off the responses to these medical risk-related questions of the SA survey, 7% constitutes a red flag that cannot be ignored.

Future research on this subject should strive to eliminate recall bias by investigating informed consent within days or hours of being counselled–even before undergoing the donation procedure; and by assessing not only egg donors’ subjective opinion about whether they understand the risks involved, but also assessing whether they objectively understand such risks [20,21].

### SA egg donors are remarkably open to identity release

Should SA’s current legal regime of anonymity be changed to one that allows donor choice regarding identity release, most SA egg donors can be expected to choose to be ‘identity-release’ egg donors. Such a legal regime would ultimately place the decision about whether the donor-conceived child would know the name of his or her donor in the hands of the legal parents, as they would be able to choose between anonymous and identity-release egg donors. Should a donor-conceived child be given the right to know his or her genetic origins, a parental choice-based system may, however, be perceived as infringing on such a right of the child, and hence would not be a viable model. In a scenario where the law was changed to provide for a right to know one’s genetic origins, a compulsory identity release model may appear to be more attractive to policy-makers. A common argument against this model is that it may cause gamete donation to decrease substantially, and hence compromise the prospective child’s likelihood of actually coming into existence. For instance, Pennings [22] argues that the legislative move toward identity release in many European countries caused a decline in donor availability. In the SA context, this potential argument against a compulsory identity release model would be contradicted by the empirical evidence–a substantial majority (79%) of respondents in the SA survey indicated they would still have donated under such a regime. This differs from studies in the UK, which found that about half of egg donors would not donate with a non-anonymous model [23,24]. In their meta-analysis of English-language research on egg donors, Purewal and Van Den Akker [10] observed that although there appears to be a cultural shift toward disclosure, there remains a sizeable minority of donors who would not donate non-anonymously. Judging by the results of the SA survey, it appears that this cultural shift is notable among SA egg donors.

### Donor remuneration is below egg donors’ expectations of fairness

The SA survey result that a significant majority (69%) of respondents perceived the current legal regime of limiting donor compensation to cost-recovery as being fair *in principle*, comes with an important caveat: The current statutory provisions that limit donor compensation to cost-recovery are not followed in practice–instead, a de facto fixed-amount system is ubiquitous. However, respondents are unlikely to be aware of the legal technical distinction between the two, and may simply think that the two are one and the same.

The SA survey shows that in the opinion of egg donors, a fair and realistic amount of compensation would be around R11,250. This result should be seen in the light that the respondents are likely to be thinking within the paradigm to which they are used, namely the de facto fixed-amount system, rather than the cost-recovery system provided for in the law. This again constitutes a limitation of this study. An increase to R11,250 –about 60% more than the current practice of paying R7,000 –would bring SA more in line with other countries that adhere to fixed-amount systems. For instance, in Portugal the fixed amount is €627 (~R10,200; 46% more than SA), in the United Kingdom it is £750 (~R14,000; 100% more than SA), and in Spain, with variations it is €900 (~R14,650; 109% more than in SA) [12]. It is interesting to note that respondents’ opinions on a fair and realistic amount of compensation is not influenced by their income, as there is no significant correlation between these two amounts.

Given the purpose of this article–to communicate and analyse the findings of the SA survey–this article does not engage in a legal critique of the de facto, fixed-amount system of egg donor compensation. However, the apparent discrepancy between the statutory provision (that donor compensation must be limited to cost-recovery), and actual practice (that donor compensation is a fixed amount, pegged to the maximum amount determined by SASREG) is a deserving subject for future legal analysis. Also, the potential confusion caused by this law–practice discrepancy constitutes a limitation of this study, which calls for making a clear distinction between the two systems of compensation (fixed amount vis-à-vis cost recovery), isolated from any potentially confusing connections with *current* law or practice.

### Conclusion

The purpose of the SA survey was to gain information from egg donors which could be pertinent to policy development on egg donation. The results generally paint a positive picture of how SA egg donors experience egg donation, but highlight two main issues that should be addressed: First, although egg donors are mainly motivated by a desire to help infertile women, compensation cannot be ignored–and against this background, egg donors feel that fair and realistic compensation should be about 60% more than the R7,000 they receive currently. This is a clear call to action for public policy actors. Second, the quality of informed consent by egg donors and feedback to egg donors after egg donation should be improved. This is primarily a call to action for the medical practitioners involved in egg donation.

In the light of current policy debate in SA on (a) whether to create a right to know one’s genetic origins, and (b) what the implications of such a right should be, the most pertinent result of the SA survey is the remarkable openness of the respondents to donating non-anonymously. It is important to note, however, that this result and its implications are not exhaustive of all arguments and policy considerations relevant to the debate on (a) and (b) above.

## Supporting information

S1 Document SASREG’s predecessor organisations’ Guidelines for Gamete Donation of 2008. (DOC)

S2 Document SASREG’s 2014 amendment (relating to egg donor compensation) of the Guidelines for Gamete Donation of 2008. (PDF)

S1 Alternative language abstract The abstract in Afrikaans. (DOCX)

S2 Alternative language abstract The abstract in Zulu. (DOCX)
