# Supplementary material for: Egg donors’ motivations, experiences, and opinions: A survey of egg donors in South Africa
Source: PLoS One. 2020 Jan 15;15(1):e0226603. doi: 10.1371/journal.pone.0226603 (PMC6961873; doi:10.1371/journal.pone.0226603)
Supplement: S1 Document — (DOC) [file pone.0226603.s001.doc]

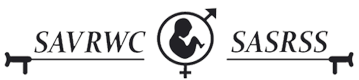


**2008 GUIDELINES FOR GAMETE DONATION**

**The Policy Committee of the South African Society of Reproductive Science and Surgery.**

***The 2008 Guidelines for gamete donation provides the latest recommendations for clinicians involved in assisted conception using gamete donors. This includes updated advice regarding the recruitment and screening of donors, management of donors and recipients and preparation of documentation necessary for clinic records according to recent legislation in South Africa.***

**CONTENTS OF THE GUIDELINES:**

- **Introduction to the guidelines**
- **Advertising for recruitment of gamete donors**
- **Age of prospective gamete donors**
- **GUIDELINES FOR SPERM DONATION**
- **Indications for sperm donation**
- **Evaluation of the recipient couple**
- **Psychological evaluation of the recipient couple**
- **Consent for the recipient couple**
- **Guidelines regarding pelvic / tubal assessment in the female recipient**
- **Selection of the sperm donor**
- **Consent for sperm donors**
- **Screening and testing of donors**
- **Payment of sperm donors**
- **Cryopreservation of sperm**
- **Limitations of donor use**
- **Sperm donor file – information to be kept in clinic records**
- **Information to be forwarded annually to Director General**
- **GUIDELINES FOR OOCYTE DONATION**
- **Indications for oocyte donation**
- **Evaluation of the recipient couple**
- **Age of the recipient female**
- **Psychological evaluation of the recipient couple**
- **Consent for the recipient couple**
- **Selection of the oocyte donor**
- **Fertility patients as oocyte sharing donors**
- **Consent for the oocyte donor**
- **Screening and testing of oocyte donors**
- **Payment of oocyte donors**
- **Limitations of donor usage**
- **Oocyte donor file – information to be kept in clinic records**
- **Information to be forwarded annually to the Director General**

**INTRODUCTION**

Gamete donation is a valuable tool for Reproductive Medicine Specialists, and forms an essential part of modern Assisted Reproductive Therapy (ART). The society recognizes that this is a complex area, and therefore regularly updated guidelines are drafted to assist members with the practice of gamete donation.

The legislation in South Africa on gamete donation is published as Chapter 8 of the National Health Act (Act no 61 of 2003)^1^ and the Regulations regarding Artificial Fertilisation and Related Matters published in the Government Gazette (no 29527 of 2007)^2^. The guidelines do not replace the legislation which is mandatory, but rather provide additional information for medical practitioners.

The American Society for Reproductive Medicine (ASRM) has published guidelines on gamete donation in 2006^3^, and the HFEA (Human Fertilization and Embryology Authority) has also published guidelines in their Code of Practice^4^. The guidelines that follow have been adapted from these sets of guidelines to be in line with best International Practice. However, the legislation and socio-economic funding of healthcare in South African are unique and therefore the guidelines are written in the South African context.

This guideline replaces the previous guideline published by the South African Society for Reproductive Science and Surgery (SASRSS) in 2004^5^.

**ADVERTISING FOR RECRUITMENT OF GAMETE DONORS**

1. Advertising and publicity materials should be designed and written with regard to the sensitive issues involved in recruiting donors.
2. Statements about “earning money” or “financial gain” should be avoided, but reference to reimbursements or compensation are acceptable.

**AGE OF PROSPECTIVE GAMETE DONORS**

1. Unless there are exceptional reasons for doing so, sperm should not be taken from donors aged 46 years or older. If there are exceptional reasons, these should be recorded.
2. Unless there are exceptional reasons for doing so, oocytes should not be taken from donors aged 36 years or older. If there are exceptional reasons, these should be recorded.
3. The minimum age for sperm and oocyte donors is 18 years (as per the National Health Act).

**GUIDELINES FOR SPERM DONATION**

**Indication for sperm donation**

1. The male partner has severe azoospermia, severe oligospermia or other significant sperm or seminal fluid abnormalities.
2. The male partner has ejaculatory dysfunction
3. The male partner has significant male factor infertility (ie significant oligoasthenospermia or prior failure to fertilize with intrauterine insemination) and the patient elects not to have the IVF/ICSI procedure.
4. The male partner has a significant genetic defect or the couple has produced a child affected by a condition that cannot be detected on genetic testing.
5. The male partner has a transmissible infection that cannot be eradicated.
6. The female partner is Rh – negative and is severely isoimmunized, and the male partner is Rh- positive.
7. Females without male partners.

**Evaluation of the recipient couple**

1. The male partner should have completed an appropriate clinical evaluation and have alternative treatments discussed with him if they are feasible.
2. HIV testing for the male partner is strongly recommended for medico-legal reasons, in case the female partner seroconverts after donor insemination. The usual tests for STIs (as for the female partner) are also recommended.
3. The female partner should have routine medical and reproductive history taken and a complete physical examination including a pelvic examination. Abnormalities detected may require more detailed evaluation and treatment prior to proceeding with insemination.
4. Recommended tests include:
   - Blood group, Rhesus factor and antibodies
   - Rubella titers. Vaccination should be offered if the individual is not immune.
   - HIV 1 and 2
   - serologic test for syphilis
   - Hepatitis B surface antigen
   - Hepatitis C antibody
   - CMV IgM. If there is evidence of recent infection (positive IgM), IgG levels can also be tested. If there is a fourfold rise in IgG levels (compared with a previous test level) or the IgM levels are at least 30% of the IgG level, then donor insemination should be postponed.
   - Additional tests which can be requested at the discretion of the clinician are Neisseria gonorhoeae, Chlamydia trachomatis and HTLV 1 and 2.
5. Women with regular cycles can be assumed to be ovulating. When doubt exists, serum day 21 progesterone levels, LH surge testing and/or follicle tracking can be performed to document ovulation.
6. Appropriate methods to time ovulation improve the success of insemination procedures.

**Psychological evaluation of the recipient couple**

The decision to proceed with donor insemination is a complex one, and patients may benefit from psychological counseling to aid in this decision. The clinician should offer psychological counseling to all couples, and require psychological consultation for couples in whom factors appear to warrant further evaluation.

**Consent for the recipient couple**

Informed consent should be taken from the patient (and her partner if applicable).

**Selection of the sperm donor**

1. The donor should be healthy and have no history of genetic abnormalities.
2. The donor should be of a legal age (18 years old) and ideally less than 40 years since there is a progressive increase in aneuploid sperm with increased age.
3. Donors with established fertility are desirable but this is not essential requirement.
4. Psychological evaluation of the sperm donor may be performed by a mental health professional at the recipient’s request.
5. It is important to ascertain whether the sperm donor understands which information about him might be disclosed and any plans that may exist relating to future contact with the offspring of his donation. The donor should decide whether a toddler or adult picture of himself can be made available to the recipient.
6. Directed or known sperm donors are encouraged to have a psychological evaluation of themselves and their partners (if applicable) prior to making the decision to proceed with sperm donation. In this circumstance the female recipient and her partner should also be evaluated by a psychologist, and the relationship between the female recipient and the sperm donor should be explored.
7. In the case of a known sperm donor, it is important that both parties receive legal advice regarding theirs rights / obligations in relation to the child conceived.
8. No owner, laboratory director or employee of a facility performing donor insemination may be a sperm donor in that practice. Neither the patient’s physician nor the individual performing the insemination can be the sperm donor.

**Consent for sperm donors**

Informed consent should be taken from the sperm donor. This should include a denial of having any of the risk factors for STIs and genetic diseases.

**Screening and testing of donors**

1. Semen analysis. It is suggested that several samples be examined prior to proceeding. In addition the semen sample should be analysed at each donation to check there are sufficient motile sperm present prior to cryopreservation.
2. Genetic evaluation. Genetic screening should be performed for heritable diseases by means of a family history questionnaire. Other genetic screening can be perfomed at the request of the recipient such as cystic fibrosis carrier status or any other test the recipient deems necessary in her specific situation. A karyotype is not necessary but may be done at the recipient’s request.
3. Personal and sexual history should be obtained to detect donors who have a high risk of STIs. Prospective donors who have a history of: sex with another man in the preceding 5 years, casual sexual relations frequently with different partners, injecting drugs for non-medical reasons, haemophilia or other coagulation disorders who have received human derived clotting factor concentrates or men who have had sex in exchange for money in the preceeding 5 years. Men who have had sex with any person in the preceeding 12 months who fits the criteria already listed of any person suspected of having HIV or Hepatitis B or C should be excluded.
4. Men who have a history of being in contact with infections diseases (HIV, Hepatitis B and C) via inoculation or having contamination into an open wound should be excluded. Men in close contact with people who have other viral hepatitis should also be excluded. Men who have a history of symptoms or a diagnosis of West Nile fever, SARS (Sudden Acute Respiratory Syndrome) or a family history of transmissible spongiform encephalopathy should be excluded. Men who have been treated for syphilis, gonorrhoea or Chlamydia within the preceeding 12 months should be excluded.
5. Men who have undergone acupuncture, body piercing or tattooing procedures within the preceeding 12 months in which sterile procedures were not used or it is not not clear whether sterile procedures were used should be excluded.
6. Men who have been incarcerated in jail in the preceding 12 months should be excluded.
7. Men who have received a xenotransplant (live cells, tissue, or organs from a non-human source) should be excluded.
8. Men who have received human organ or tissue transplant should be excluded.
9. Physical examination. Donors should have a physical examination to check for the presence of urethral discharge, genital warts and ulcers. They should be excluded if any of these are discovered.
10. Laboratory testing. The following tests should be done on donors: HIV type 1 and 2, Hepatitis B Surface Antigen, Hepatitis C Ab, syphilis serology. In addition the following tests can be done if the recipient requests these (as per ASRM guidelines): HTLV 1 and 2, CMV IgM and IgG, neisseria gonorrhoea, Chlamydia.
11. Known or directed donors should have the same testing / evaluation as anonymous donors.
12. The donor must disclose whether he has donated previously in any other fertility unit. This is important so that the total number of pregnancies obtained from his donated sperm can be ascertained.

**Cryopreservation of sperm**

It is recommended that all sperm for donation is cryopreserved prior to use in order to detect transmissible disease in the early “window period”. The Regulations of the National Health Act stipulate that the donor should be tested for STIs on two occasions not less than 3 months apart and with one of the tests done within one month of the donated sample.

**Payment of sperm donors**

The donor can be reasonably compensated for his time and expenses.

**Limitations of donor use**

The Regulations for the National Health Act state that donor sperm may not be used in the following situations:

- where five children exist who have been conceived from artificial fertilization using his gametes
- where 2 or more pregnancies exist as a result of artificial fertilization using his gametes
- the possibility exists that after the intended artificial fertilization procedure, more than two pregnancies may exist simultaneously as a result of the artificial fertilization with the donor’s gametes
- representation can be made to the National Department of Health can be made to use the donor sperm outside of the above regulations.

**Sperm donor file – information to be kept in clinic records**

The following information should be kept in the clinic’s donor file:

- Full name, surname, date of birth and identity number
- A unique donor identification number
- Age, height, mass, eye colour, hair colour, complexion, population group, nationality, sex, religion, occupation, highest educational qualification and fields of interest
- Family history
- Donor’s wishes in relation to how many donations his gametes may be used for
- Psychological evaluation of the donor if performed
- Informed consent form
- Results of investigations (test results for STIs, genetic tests and semen analysis)
- Number of donation performed with the donor’s sperm and the dates of these donations.
- Number of children conceived from the donor

The sperm donor file should not be made available to any other person except in terms of legislation or a court order.

The donor file should not be destroyed, except with the written permission of the Director General of Health.

**Information to be forwarded annually to Director General**

It is the responsibility of the Director General of Health to establish a central electronic data bank to record the details of gamete donors.

The following information regarding the gamete donor should be provided at the request of the Director General of Health prior to the 31 January each year (for the preceding year):

- The unique identification number of the gamete donor file
- The number of donations, and the dates of the donations
- The number of children conceived through the process of artificial fertilization that have been born alive from gametes of the donor

Inform the Director General of donors where five children have been born from a gamete donor, and then destroy the remaining gametes of that donor and make a note in the clinic donor file.

**GUIDELINES FOR OOCYTE DONATION**

**Indications for oocyte donation**

1. Women with hypogonadotrophic hypogonadism
2. Women of advanced reproductive age
3. Women who have reduced ovarian reserve or ovarian dysfunction / failure
4. Women who are known to be the carrier of a significant genetic defect or who have a family history of a condition where the carrier status cannot be determined
5. Women with poor oocyte or embryo quality or multiple failures during prior attempts to conceive with assisted reproductive technologies.

**Evaluation of the recipient couple**

1. Medical and reproductive history.
2. Complete physical examination including a pelvic examination
3. Assessment of the uterine cavity. This can be done by hysteroscopy, hysterosalpingogram (HSG) or saline sonography.
4. Blood tests for both recipients partners (if applicable):
   - Blood group, Rhesus factor and antibodies (female recipient only)
   - Rubella titers. Vaccination should be offered if the individual is not immune. (female recipient only)
   - HIV 1 and 2
   - serologic test for syphilis
   - Hepatitis B surface antigen
   - Hepatitis C antibody
   - CMV IgM. If there is evidence of recent infection (positive IgM), IgG levels can also be tested. If there is a fourfold rise in IgG levels (compared with a previous test level) or the IgM levels are at least 30% of the IgG level, then oocyte donation treatment should be postponed.
   - Additional tests which can be requested at the discretion of the clinician are Neisseria gonorhoeae, Chlamydia trachomatis and HTLV 1 and 2.

**Age of the recipient female**

Since oocyte donation treatment can be performed on older post-menopausal women, special consideration should be given to couples where the female recipient’s age is greater than 50 years. Women over the age of 55 years should be discouraged from oocyte donation programs.

It is recommended that the following extra steps are followed in these situations: psychological review of the recipients by a mental health professional, medical assessment by the obstetrician who will be providing antenatal care with regards to the pregnancy risks, a physician’s assessment of the female recipient’s general health with specific regards to the presence of hypertension, diabetes or coronary heart disease. A stress ECG and mammogram should be performed.

Once the above information is available, the clinician should be present the case for discussion at an Ethics committee comprising a multi disciplinary team of specialists.

**Psychological evaluation of the recipient couple**

The decision to proceed with oocyte donation is a complex one, and patients may benefit from psychological counseling to aid in this decision. The clinician should offer psychological counseling to all couples, and require psychological consultation for couples in whom factors appear to warrant further evaluation.

**Consent for the recipient couple**

Informed consent should be taken from the female recipient (and her partner if applicable).

**Selection of the oocyte donor**

1. Anonymous vs known oocyte donors. Pragmatic considerations such as the difficulty in recruiting suitable donors support the use of known oocyte donors in the appropriate clinical situations.
2. Psychological evaluation by a psychologist should be performed on the donor and her partner (if applicable). The potential impact of any relationship between the oocyte donor and the recipient should be explored. The potential psychological risks of donating oocytes should be discussed and it should be ascertained whether the donor understand all the implications and risks of the procedure. The donor should be informed as to the extent to which information about her may be disclosed and any plans that exist regarding future contact with the child. Donors should be of legal age (18 years) and preferably between the ages of 20 and 35 years.
3. Donors less than 21 years should have psychological evaluation by a psychologist and the decision to proceed should be determined on an individual basis.
4. If a prospective donor is greater than 34 years, then the recipients should be informed about the increased risks for aneuploidy and the effect of donor age on success.
5. Proven fertility in the donor is desirable but not essential.
6. The donor should be healthy have no history of genetic abnormalities. Genetic evaluation. The donor’s family history should be carefully recorded by means of questionnaire to exclude any history of heritable disease. In addition, the recipient can request the following genetic tests which are of value: cystic fibrosis carrier status, karyotype of the donor, Fragile X mutation in the donor or any other test the recipient deems necessary in her specific situation.
7. No owner, laboratory director or employee of a facility performing oocyte donation may be an oocyte donor in that practice.
8. If an agency is used to recruit donors, no individual that has a financial interest in that agency may be used as an oocyte donor.

**Fertility patients as oocyte sharing donors**

If sharing of oocytes is contemplated, informed consent must be obtained prior to the start of the cycle of retrieval. The conditions governing the sharing of oocytes should be specified in advance.

The evaluation of the patient should be the same as any other oocyte donor including the psychological assessment, medical examination and investigations specified under the screening and testing of oocyte donors section.

**Consent for the oocyte donor**

Informed consent should be taken from the oocyte donor. This should include a denial of having any of the risk factors for STIs and genetic diseases.

The medical risks of the procedure (ovarian hyperstimulation and other risks) should be clearly explained to the donor.

**Screening and testing of oocyte donors**

1. Donors should be healthy and have no history of heritable diseases.
2. Genetic evaluation. Genetic screening should be performed for heritable diseases by means of a family history questionnaire. Other genetic screening can be perfomed at the request of the recipient such as cystic fibrosis carrier status or other genetic illnesses that may be present in the recipient’s family history. A karyotype and Fragile X test may be useful tests for oocyte donors.
3. Personal and sexual history should be obtained to detect donors who have a high risk of STIs. Prospective oocyte donors who have a history of: casual sexual relations frequently with different partners, injecting drugs for non-medical reasons, haemophilia or other coagulation disorders who have received human derived clotting factor concentrates or donors who have had sex in exchange for money in the preceeding 5 years. Oocyte donors who have had sex with any person in the preceeding 12 months who fits the criteria already listed of any person suspected of having HIV or Hepatitis B or C should be excluded.
4. Oocyte donors who have a history of being in contact with infections diseases (HIV, Hepatitis B and C) via inoculation or having contamination into an open wound should be excluded. Donors in close contact with people who have other viral hepatitis should also be excluded. Donors who have a history of symptoms or a diagnosis of West Nile fever, SARS (Sudden Acute Respiratory Syndrome) or a family history of transmissible spongiform encephalopathy should be excluded. Oocyte donors who have been treated for syphilis, gonorrhoea or Chlamydia within the preceeding 12 months should be excluded.
5. Oocyte donors who have undergone acupuncture, body piercing or tattooing procedures within the preceeding 12 months in which sterile procedures were not used or it is not not clear whether sterile procedures were used should be excluded.
6. Oocyte donors who have been incarcerated in jail in the preceding 12 months should be excluded.
7. Oocyte donors who have received a xenotransplant (live cells, tissue, or organs from a non-human source) should be excluded.
8. Oocyte donors who have received human organ or tissue transplant should be excluded.
9. Physical examination. Oocyte donors should have a physical examination including a pelvic examination prior to donating.
10. Psychological evaluation by a registered psychologist or mental health professional is strongly recommended.
11. Laboratory testing. The following tests should be done on oocyte donors: HIV type 1 and 2, Hepatitis B Surface Antigen, Hepatitis C Ab, syphilis serology and CMV IgM. In addition the following tests can be done if the recipient requests these (as per ASRM guidelines): HTLV 1 and 2, CMV IgG, neisseria gonorrhoea, Chlamydia, blood group, Rhesus status.
12. Known oocyte donors or egg sharing donors should have the same testing / evaluation as anonymous donors.
13. The donor must disclose whether she has donated previously in any other fertility unit. This is important so that the total number of pregnancies obtained from her gametes can be ascertained.

**Payment of oocyte donors**

Monetary compensation of the donor should reflect the time, inconvenience, financial costs to the donor – eg. travel, loss of income and childcare costs, physical and emotional demands and risks associated with oocyte donation and should be at a level that minimizes the possibility of undue inducement of donors and the suggestion that payment is for the oocytes themselves.

The monetary compensation should not be predicted on the clinical outcome (no of oocytes or pregnancy outcome) but rather on fair compensation for the procedure of donating eggs.

At the time of writing this guideline, it is the society’s opinion that monetary compensation for an oocyte donor should be approximately 5000 - 6000 rand. Compensation amounts in excess of 10 000 rand should only be paid in exceptional circumstances.

Donors should receive financial compensation via fertility clinics.

**Limitations of oocyte donor use**

The Regulations for the National Health Act state that donor oocytes may not be used in the following situations:

- where five children exist who have been conceived from artificial fertilization using her gametes
- where 2 or more pregnancies exist as a result of artificial fertilization using her gametes
- the possibility exists that after the intended artificial fertilization procedure, more than two pregnancies may exist simultaneously as a result of the artificial fertilization with the donor’s gametes

If a recipient has a child from oocyte donation and requests another oocyte donation procedure in order to have a sibling child from the same genetic donor, where the donor has reached her quota of 5 pregnancies, representation can be made to the Department of Health for permission to use the oocytes again for that recipient.

**Oocyte donor file – information to be kept in clinic records**

The following information should be kept in the clinic’s donor file:

- Full name, surname, date of birth and identity number
- A unique donor identification number
- Age, height, mass, eye colour, hair colour, complexion, population group, nationality, sex, religion, occupation, highest educational qualification and fields of interest
- Family history
- Donor’s wishes in relation to how many donations her gametes may be used for
- Psychological evaluation of the donor
- Informed consent form
- Results of investigations (test results for STIs, genetic tests)
- Number of donation performed with the donor’s oocytes and the dates of these donations.
- Number of children conceived from the donor

The oocyte donor file should not be made available to any other person except in terms of legislation or a court order.

The donor file should not be destroyed, except with the written permission of the Director General of Health.

**Information to be forwarded annually to Director General**

It is the responsibility of the Director General of Health to establish a central electronic data bank to record the details of gamete donors.

The following information regarding the gamete donor should be given on request to the Director General of Health prior to the 31 January each year (for the preceding year):

- The unique identification number of the gamete donor file
- The number of donations, and the dates of the donations
- The number of children conceived through the process of artificial fertilization that have been born alive from gametes of the donor

Inform the Director General of donors where five children have been born from a gamete donor, and then destroy the remaining gametes of that donor and make a note in the clinic donor file.

**REFERENCES**

1. National Health Act (act number 61 of 2003), published in government gazette (2003).
2. R.8 Regulations regarding Artificial Fertilization and Related Matters. Published in Government Gazette no. 29527
3. 2006 Guidelines for gamete and embryo donation. *Fertil Steril* 2006; vol 86 (suppl 4)
4. HFEA Code of Practice Booklet (As updated on November 2007)
5. Guidelines for egg donation: SASRSS. Published by SASRSS in December 2004.
