# Supplementary material for: Egg donors’ motivations, experiences, and opinions: A survey of egg donors in South Africa
Source: PLoS One. 2020 Jan 15;15(1):e0226603. doi: 10.1371/journal.pone.0226603 (PMC6961873; doi:10.1371/journal.pone.0226603)
Supplement: S2 Document — (PDF) [file pone.0226603.s002.pdf]

SASREG

Southern African Society for Reproductive Medicine and Gynaecological Endoscopy

www.fertilitysa.org.za

25 November 2014

EXECUTIVE COMMITTEE:

President
Dr P le Roux
Vice-President
Dr M Jacobson
Honorary Secretary
Ms V Wolf
Honorary Treasurer
Dr P Dalmeyer
Members

Dr D Botha
Dr A deBruin
Prof S Dyer
Ms L Els-Smit
Prof T Kruger
Dr T Matsaseng
Prof I Siebert
Dr C Venter

SECRETARIAT:

Turners Conferences (Pty) Ltd
PO Box 1935
Durban 4000
South Africa

Tel: +27 31 368 8000
Fax: +27 31 368 6623
Email: sasrss@fertilitysa.org.za

A Member of

IFFS

International Federation of Fertility Societies

Dear SASREG members,

AMENDMENT TO GAMETE DONATION GUIDELINES OF 2008
EGG DONOR COMPENSATION

Following a SASREG Committee meeting on the 22 November 2014, it was recommended that the SASREG Gamete Donation Guidelines of 2008 be updated to the following:

Egg donors should not be compensated more than R 7 000.00 per procedure from the 1 January 2015.

The previous guideline from 2008 stated that the egg donor should not be compensated more than R 5 000.00

Yours sincerely

PAUL LE ROUX
President of SASREG

On behalf of committee
