# Supplementary material for: Egg donors’ motivations, experiences, and opinions: A survey of egg donors in South Africa
Source: PLoS One. 2020 Jan 15;15(1):e0226603. doi: 10.1371/journal.pone.0226603 (PMC6961873; doi:10.1371/journal.pone.0226603)
Supplement: S1 Alternative language abstract — (DOCX) [file pone.0226603.s003.docx]

Abstract in Afrikaans

**Opsomming**

Die doel van hierdie studie was om ’n wye verskeidenheid van kwessies aangaande eiersel-skenking in Suid-Afrika te ondersoek. Die studie was gedoen op eiersel-skenkers in die databasis van ’n Kaapstadse eiersel-skenkings-agentskap wat binne ’n jaar voor die studie geskenk het. 150 eiersel-skenkers uit ’n bevolking van 226 het deelgeneem aan ’n aanlyn meningsopname. Die hoofresultate is as volg: 95% van die respondente het eiersel-skenking as positief ervaar. Nietemin, 7% van die respondente rapporteer dat hulle nie behoorlik ingeligte instemming gegee het nie, en dieselfde persentasie van respondente rapporteer dat hulle nie weet of enige mediese risiko’s werklik gematerialiseer het as gevolg van hulle donasies nie. Dit is ’n bron van kommer, en behoort verder ondersoek te word. Ten opsigte van skenker-anonimiteit, wat tans die regsposisie in Suid-Afrika is: 79% van respondente het aangedui dat hulle steeds sou geskenk het, selfs indien hulle regtens verplig sou wees om hulle identiteite bekend te maak. Dus, dit blyk onwaarskynlik dat regshervorming weg van die huidige stelsel van skenker-anonimiteit ’n wesenlike impak op die toevoer van skenker-eierselle sal hê. Ten opsigte van motivering rapporteer respondente dat hulle primêr gemotiveer is deur deernis. Desnieteenstaande glo respondente dat ’n billike en realistiese vergoedingsbedrag omtrent 60% hoër is as die bedrag wat tans betaal word as ’n nasionale standaard vaste vergoedingsbedrag. Hierdie vaste-vergoedingsbedrag-stelsel se wettigheid, impak op skenker-profiel, en bedrag-grootte verdien verdere ondersoek.
