# Supplementary material for: Egg donors’ motivations, experiences, and opinions: A survey of egg donors in South Africa
Source: PLoS One. 2020 Jan 15;15(1):e0226603. doi: 10.1371/journal.pone.0226603 (PMC6961873; doi:10.1371/journal.pone.0226603)
Supplement: S2 Alternative language abstract — (DOCX) [file pone.0226603.s004.docx]

Abstract in Zulu

**Isifinqo**

Injongo yalesi sifundo ukuphenya izinkinga ezihlukahlukene ezixhumane nokuphisana kwamaqanda ephuma esibelethweni sabantu besifazane eNinginzimu Afrika. Loluphenyo lenziwe ebantwini besifazane abaphisana ngamaqanda abo ababhaliwe esizindeni semininingwane ye nhlangano yokuphisana kwamaqanda yase-Cape Town onyakeni owawudlule ngaphambi kwalulophenyo. Abantu besesifazane abangu-150 ebantweni abawu-226 ababaphisane ngamaqanda abo baphendule amaphepha anezihlelo zembizo ezikwi-inthanethi. Imiphumela ebalulekile yilezi: abaphenduli abangu-95% bazizwe ngathi ukuphisana kwamaqanda abo kuhambe kahle. Nokho, abaphenduli abangu-7% bathi abazange benze isinqumo sabo emva kokwaziswa ngedlela efanele, nabanye, ngokuningi okucishe kulingane, bathe abazange baziswe ukhuthi kukhona yini okubavelelile phakathi kwezingozi ezimpilweni zabo ezihlangane nokophisana kwamaqanda. Lokhu kuyakhathaza futhi kumele kuphenywe kwabanzi. Mayelana nokufihlwa yeminingwane yabantu abaphisana ngamaqanda abo, okulona uhlelo lomthetho eNingizimu Afrika, abaphenduli abangu-79% bathe ngabe baqhubekile nokuphisana ngamaqanda abo ngisho kuthiwe umthetho bubaphoqa ukuthi bambule ukuthi bangobani. Ngakho-ke, uma kwenzeke ushintshe lomthetho, akubukeki ngathi lokhu kungathinta iningi yabantu abaphisana ngamaqanda abo. Mayelana nezizathu zabo, abaphenduli bathe uzwelo ulona elibenza baphisane ngamaqanda. Noma kunjalo, abaphenduli bakholwa ukuthi inkokhelo abayitholayo ingaba nobuqhoto neqiniso uma ingaba-60% ngaphezu kwalokhu abakutholayo njengamanje, okusezingeni esingaguquki esizweni lonke. Lesi simo sezinga yenkokhelo engaguquki kumele iphenywe kwabanzi ekutheni isitmthethweni yini, ukuthi ina mphumelo muni ebantwini abaphisana ngamaqanda, nokuthi iyumali afanele yini.
